# Multi-Focus Image Fusion for Full-Field Optical Angiography

**DOI:** 10.3390/e25060951

**Published:** 2023-06-16

**Authors:** Yuchan Jie, Xiaosong Li, Mingyi Wang, Haishu Tan

**Affiliations:** 1 Shien-Ming Wu School of Intelligent Engineering, South China University of Technology, Guangzhou 510640, China; jyc981214@163.com; 2 School of Physics and Optoelectronic Engineering, Foshan University, Foshan 528225, China; wangmingyi@mail.bnu.edu.cn

**Keywords:** full-field optical angiography, nonsubsampled contourlet transform, image fusion, contrast spatial frequency, sparse representation

## Abstract

Full-field optical angiography (FFOA) has considerable potential for clinical applications in the prevention and diagnosis of various diseases. However, owing to the limited depth of focus attainable using optical lenses, only information about blood flow in the plane within the depth of field can be acquired using existing FFOA imaging techniques, resulting in partially unclear images. To produce fully focused FFOA images, an FFOA image fusion method based on the nonsubsampled contourlet transform and contrast spatial frequency is proposed. Firstly, an imaging system is constructed, and the FFOA images are acquired by intensity-fluctuation modulation effect. Secondly, we decompose the source images into low-pass and bandpass images by performing nonsubsampled contourlet transform. A sparse representation-based rule is introduced to fuse the lowpass images to effectively retain the useful energy information. Meanwhile, a contrast spatial frequency rule is proposed to fuse bandpass images, which considers the neighborhood correlation and gradient relationships of pixels. Finally, the fully focused image is produced by reconstruction. The proposed method significantly expands the range of focus of optical angiography and can be effectively extended to public multi-focused datasets. Experimental results confirm that the proposed method outperformed some state-of-the-art methods in both qualitative and quantitative evaluations.

## 1. Introduction

Blood flow reflects the health status of biological tissues to some extent, and blood flow imaging plays a vital role in clinical diagnosis and treatment. Different physiological mechanisms can be discovered and identified in advance by understanding overall changes in the structure and function of an organism’s microcirculation. Two common bio-optical properties are relevant in practical imaging applications for living creatures, such as those involving zebrafish tissue, mouse ears, and the human retina. First, light scattering suffers from a lack of intensity as a result of weakly scattering samples and near-transparent media; second, the spatial distribution of capillaries with a laminar structure is not intricate. The surfaces of these biological samples are uneven, and their thicknesses are approximately 1 to 5 mm, which makes obtaining distinct long-depth-of-field angiographic images using a lens with a large magnification relatively difficult. Generally, global blood flow conditions can be continuously reflected by taking advantage of full-field and high-resolution imaging instruments combined with full-field optical imaging methods, an approach that has been shown to improve the reliability of biological research.

Full-field optical angiography (FFOA) is a rapidly developing vascular imaging technique with high spatial and temporal resolution. FFOA is appropriate for real-time imaging of living creatures and has a wide range of applications in both bioscience research and clinical diagnosis. In recent years, various optical imaging methods have emerged [1,2], such as full-field optical coherence tomography (FF-OCT) [3], laser scatter contrast imaging (LSCI) [1], and two-dimensional visualization using full-field laser Doppler imaging (LDI) [2]. These imaging methods can improve the resolution, imaging speed, and sensitivity of bio-optical imaging to some extent and can effectively image the functionality and structure of biological tissues. Unfortunately, they have a common drawback in that only vascular information in a plane within the depth of field can be acquired. To solve the defocus problem caused by the uneven surfaces and thicknesses of biological samples, as well as the limitations of the depth of field of imaging cameras, images with diverse focus regions must be integrated to obtain an FFOA image with a long depth of field. Multi-focus image fusion is one feasible way to address this issue.

Multi-focus image fusion exploits specialized fusion algorithms to integrate multiple source images containing different focus regions in the same scene to obtain a fused image [4,5,6,7,8], which provides a more comprehensive, objective, and thorough interpretation of a scene compared to partially focused source images. Multi-focus image fusion methods for biomedical imaging can be broadly classified into four categories, including spatial domain (SD)-based methods [9,10,11], multi-scale transform (MST)-based methods [7,12,13,14], deep learning (DL)-based schemes [15,16,17,18,19], and sparse representation (SR)-based schemes [20,21,22,23,24].

SD-based methods perform focus detection on pixels, blocks, or regions of source images in the spatial domain directly and then combine the selected focused pixels to generate fusion results. Jie et al. [25] proposed using difference-of-Gaussians to detect salient edges of source images to effectively preserve details. Zhang et al. [9] designed a joint guided image filtering-based approach, and a combination of static and dynamic guidance was employed in a joint guided image filter to recognize and extract important features. Ma et al. [10] designed an algorithm based on a random-walk estimation, which achieved improved estimation results at the expense of greater running time. Liu et al. [11] proposed an image fusion scheme utilizing a multi-scale shearing nonlocal guided averaging filter with shift-invariance to express the structure of an image sparsely. However, the fused results produced by SD schemes are highly dependent on the accuracy of pixel activity detection. If the selection of pixels is unreasonable, suboptimal results will be obtained. This may be attributed to two principal causes of SD schemes: First, the determination of the focus of smooth areas in the focus region is error-prone, and second, block effects or blurring may occur at the focus boundary [26].

DL-based schemes are impressive for numerous tasks, such as person reidentification, fault recognition, and image denoising. With the development of image fusion technology, DL has also been applied to image fusion. Amin-Naji et al. [16] proposed an approach based on CNN with integrated learning for increased variety between models and datasets to reduce the occurrence of overfitting to some extent. To reduce the loss of details of the source images, Zhang et al. [17] provided an image-fusion scheme using joint adaptive and gradient constraints. Xu et al. [18] developed an unsupervised fusion network to automatically estimate vital pixels using information measurements and feature extraction. Ma et al. [19] developed a fusion method based on the Swin transformer to preserve the details and structure of source images. Although DL-based methods enhance the quality of fused images to a certain degree, the lack of suitable datasets and the challenge of designing a suitable loss function could constrain the fusion performance.

Unlike the SD-based and DL-based approaches, MST techniques transform images into a given transform domain at the beginning of the process, merge the transformed coefficients according to certain fusion rules, and finally reconstruct the fused image by performing an inverse transform on the post-fusion coefficients. Popular decomposition tools include nonsubsampled contourlet transform (NSCT) [27], nondownsampled shearlet transform (NSST) [28], and so forth. NSCT is shift-invariant, which can avoid the appearance of pseudo-Gibbs phenomena. Xiao et al. [14] employed a Hessian matrix with different scales to decompose source images into feature and background regions. The extremum of the determinant of the Hessian matrix plays a vital role in the detection of feature regions. Li et al. [29] utilized the multiscale detail enhancement technique to preprocess the training images and fused images based on SR. Jie et al. [30] proposed a scheme based on adaptive energy-choosing technology to fuse tri-modal medical images, which can effectively detect the brightness of the cartoon layer. Li et al. [31] developed a neighbor energy activity operator to fully preserve the color in the source images. Li et al. [32] introduced the embedding bilateral filter in least squares to decompose images and utilized the salient detection method to fuse detail layers. Liu et al. [13] designed a fusion framework based on NSCT which effectively combined MST with sparse domains. Li et al. [7] put forward a residual removal technique that combined the advantages of NSCT and multi-scale focus detection to produce the fused results. Hence, we can conclude that NSCT is highly suitable for multi-focus image fusion owing to its multi-scale, multi-directional, anisotropic, and shift-invariant properties. Unfortunately, the fused image will lose some useful information, due to the process of muti-scale decomposition and reconstruction of MST-based schemes.

SR-based schemes can effectively represent the human visual system through the sparse coding mechanism. Xing et al. [20] proposed a fusion approach taking advantage of the Taylor expansion to decompose images, which fully considers the properties of the entire image. In [21], Li et al. developed a joint fusion and super-resolution scheme by designing low-rank and sparse dictionaries. Wang et al. [22] developed the multi-scale SR-based image fusion scheme in gradient domain. Although these methods [20,21,22] elevate the fusion performance, much room for improvement still exists. As to SR-based schemes, it is a big challenge to train a redundant dictionary. Moreover, its efficiency needs to be further investigated. Table 1 provides a summary of work related to multi-focus image fusion.

However, with the current multi-focus image fusion methods it is difficult to maintain the contrast, gradient, and detail information of the source images at the same time. Meanwhile, they are primarily designed based on conventional multi-focus datasets and may not be suitable for fusing FFOA images. This can be attributed to two primary causes: Firstly, scattering may occur in angiographic images, causing overlaps in the focus regions. Secondly, traditional fusion methods may produce artifacts owing to the complex shape of the focus boundary.

To overcome these two problems and obtain a long depth of field of FFOA images of high spatial and temporal resolution, in this study a novel fusion method is proposed in the NSCT domain to contribute to biological research and clinical disease diagnosis. It is worth noting that the design of fusion algorithms is usually carried out directly on existing public images in the current popular fusion methods, with little attention paid to the construction and of the imaging system as well as the acquisition of the source images. However, the production of data sources is closely linked to practical applications. Therefore, the far-reaching significance of building an imaging system based on the modulation effect of intensity fluctuations cannot be overlooked. In the design of our fusion algorithm, we utilized the NSCT to decompose the source images into low- and bandpass images. For low-pass images, SR is introduced as a popular image presentation tool, and the L1-max rule is employed to fuse the SR coefficients. Motivated by [33], a new contrast spatial frequency (CSF) rule is proposed for bandpass images to detect significant gradient features effectively.

The main contributions of this study are summarized as follows.

(1) An FFOA imaging system based on the absorption intensity fluctuation modulation effect was constructed to obtain multi-focus source images;
(2) A novel FFOA image fusion method based on SR and CSF in the NSCT domain was developed. A contrast-based rule is proposed to fuse bandpass images, which can effectively detect the structural information of FFOA images;
(3) The proposed method can effectively extend the depth of field of FFOA images and address the partial defocus problem of FFOA images caused by uneven surfaces and the varying thicknesses of biological samples. At the same time, it can also be extended to the public data sets and surpasses some state-of-the-art approaches in terms of subjective and objective evaluations.

The remainder of this study is organized as follows. Section 2 describes the construction of the optical system and the acquisition of source images. The proposed fusion method is described in detail in Section 3. In Section 4, experimental evaluation of the proposed approach is presented, including the parameter settings and the comparisons of the fusion results. Finally, the conclusions of this work are presented in Section 5.

## 2. Proposed Imaging System

**Figure 1:**
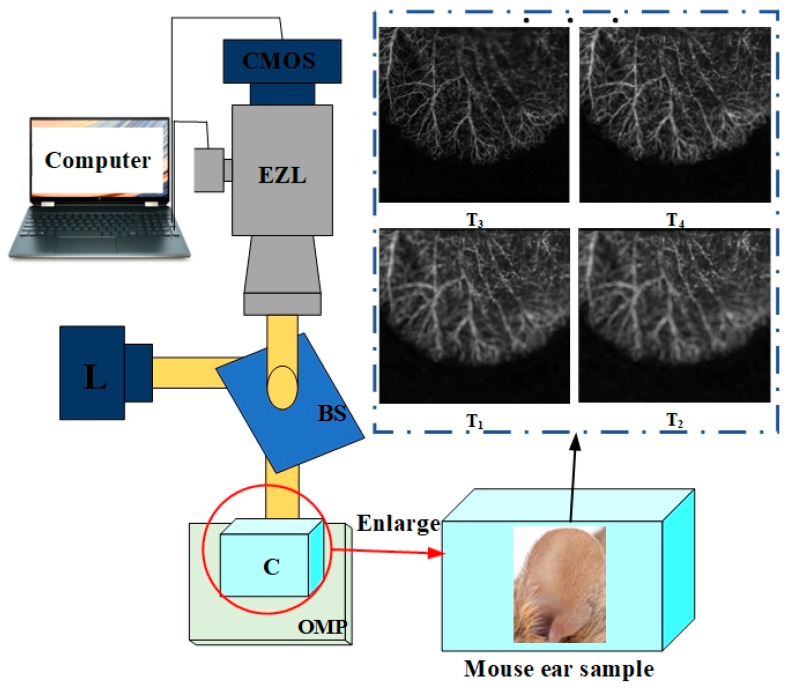
F Imaging system. Mouse ear samples were fixed on the OMP in the experiments. *T_t_* (*T_t_* = 1,2, …, *χ*) represents multiple multi-focus source images. C denotes the container, L denotes the light source, and EZL denotes the electric zoom lens.

We constructed an optical system to generate the multi-focus FFOA images by using intensity fluctuation modulation; the angiography imaging system is shown in Figure 1. Fixed samples were then moved directionally on a 2D optical moving platform (OMP). The system used a high-power laser diode L with low working current to illuminate the sample. The device L was an HL6366DG/67DG unit produced by Opnext, Fremont, CA, USA, with a bandwidth of 10 nm, a wavelength $\lambda_{0}$ of 642 nm, and a power of 80 mW. Direct lighting was used as the light source. The CMOS camera (acA2000-340 km, Basler, Exton, PA, USA) acquired signals reflected from the biological samples through a beam splitter (BS) and an electric zoom lens (EZL) and then transmitted them to a computer. The movement of the EZL was controlled by a computer to acquire multi-depth-of-field FFOA images $T_{t}$ (*t =* 1, 2, *…*, χ), where χ is the total number of images acquired by varying the focus position stepwise in increments of 0.3 mm.

Under irradiation by a light source with low coherence, the absorption coefficients of erythrocytes are far greater than those of the background, such as water and fat. As erythrocytes move through blood vessels discontinuously, they produce transient intensity fluctuations. Blood flow information is dynamic and can be captured by high-frequency signals, whereas background organizational information is static and can be represented by low frequencies. Furthermore, the high-frequency signal produced by the motion of erythrocytes can be recorded in real-time using a camera. However, because the tissue does not exhibit any momentary intensity fluctuations, only a DC signal is generated; this is known as the fluctuation modulation effect. Using this effect allows the lateral velocity of the blood to be measured accurately. The dynamic scattering speckle signal of the erythrocytes ($I_{R}$) was separated from the static scattering speckle signal ($I_{B}$) in the frequency domain. The original signal in the time domain is converted into the frequency domain using a fast Fourier transform, and the static scattering speckle signal of the erythrocytes and tissue background were obtained by high- and low-pass filtering, respectively, as expressed by Equation (1).

(1) $$\left\{\begin{array}{c} I_{R}(x,y,t)=HF[I(x,y,f)]\\ I_{B}(x,y,t)=LF[I(x,y,f)] \end{array}\right.$$

where HF[⋅] and LF[⋅] represent high- and low-pass filtering operations, respectively, and (x,y,t) denotes the space-time coordinates of a pixel at a given time *t*. The concentration of floating erythrocytes σ can be expressed as given in Equation (2):

(2) $$\sigma =\frac{n_{r}}{n_{r}+n_{b}}\approx \frac{n_{r}}{n_{b}}=\frac{I_{r}}{I_{b}}$$

where $n_{r}$ and $n_{b}$ denote the number of floating erythrocytes and the concentration of scattered particles in the background, respectively. Equation (2) indicates that the light intensity is proportional to the number of scattered particles, i.e., $I_{r}\propto n_{r}$ and $I_{b}\propto n_{b}$, and when $n_{r}$ is far smaller than $n_{b}$, the “≈” in Equation (2) is satisfied.

Average modulation depth (*AMD*) [34] is associated with erythrocyte motility and can be expressed as

(3) $$AMD(x,y)=\frac{\bar{I_{r}}(x,y)}{\bar{I_{b}}(x,y)}$$

where $\bar{I_{r}}(x,y)$ and $\bar{I_{b}}(x,y)$ are the average absolute values of the dynamic and static signals, respectively. Angiographic images produced by *AMD* calculations are called FFOA images, and the multi-focus source images used in this study fall into this category.

## 3. Proposed FFOA Fusion Model

Owing to its flexible multi-scale and multi-directional characteristics as well as to its shift-invariance, NSCT is suitable for multi-focus image fusion problems, especially because it can effectively decrease the boundary effect. Therefore, the proposed method was designed in the NSCT domain. In the following discussion, the fusion of only two source images at once is considered. However, it is important to note that the proposed method can easily be extended to cases involving more than two source images. Here, *A*, *B*, are the source images, and (*i*, *j*) denotes the pixel coordinates. The image *X* (*X* = *A*, *B*) is decomposed by NSCT to generate a low-pass image $L_{X}^{Q}$ and a series of bandpass images $H_{X}^{u,k}$ in the *u-*th layer and *k-*th direction. Here, 1≤u≤Q and 1≤k≤K, where Q and *K* represent the maximum number of decomposition layers and maximum number of directions, respectively.

**Figure 2:**
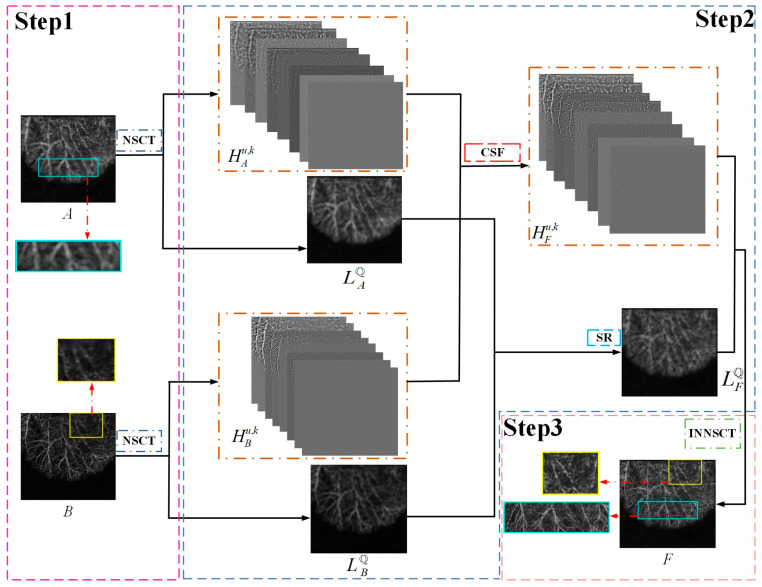
Schematic diagram of the proposed multi-focus image fusion method.

In addition, the proposed approach includes fusion rules based on SR for low-pass images and fusion rules for CSF for bandpass images. A schematic of the proposed method is shown in Figure 2. Step 1 represents the decomposition of the source images, Step 2 represents the implementation of the fusion rules, and Step 3 represents the reconstruction process.

### 3.1. Fusion of Low-Pass Images

Low-pass images contain energy and part of the weak structural details of the source image and can be approximately considered as a blurred version of the source image. The traditional rules of weighted averaging and absolute-max selection tend to reduce contrast and lose details, resulting in suboptimal fusion results. SR uses a flexible linear representation of an image through a redundant dictionary that can effectively express the intrinsic structural features of an image. Furthermore, the *l*_1_-max rule is applied to the sparse coefficients to preserve the energy and part of the detail information of source images. Recently, SR has also been widely used in super-resolution image reconstruction [23] and person reidentification [35].

Suppose that $L_{A}^{Q}$ and $L_{B}^{Q}$ denote the low-pass images of *A* and *B*. The developed low-pass image-fusion rule includes the following three steps.

Step a: Block extraction and vectorization

The sliding window technique is used to divide $L_{A}^{Q}$ and $L_{B}^{Q}$ into 8 × 8 blocks of the same size from top to bottom and left to right, with overlapping λ pixels between the blocks, i.e., the step length. *W* blocks corresponding to $L_{A}^{Q}$ and $L_{B}^{Q}$ are obtained in this manner, and the set of image blocks can be represented as $\{p_{L_{A}}^{l}{\}}_{l=1}^{w}$ and $\{p_{L_{B}}^{l}{\}}_{l=1}^{w}$. Then, each image block is pulled into a column vector, i.e., $p_{L_{A}}^{l}\to {\vec{q}}_{L_{A}}^{l}$ and $p_{L_{B}}^{l}\to {\vec{q}}_{L_{B}}^{l}$.

Step b: Sparse coding

The orthogonal matching pursuit algorithm [36] is employed to determine the SR coefficients $\left\{v_{L_{A}}^{l},v_{L_{B}}^{l}\right\}$ of $\left\{{\vec{q}}_{L_{A}}^{l},{\vec{q}}_{L_{B}}^{l}\right\}$.

(4) $$v_{L_{A}}^{l}=\underset{v}{argmin}{\left\|v\right\|}_{0}s.t.{\left\|{\vec{q}}_{L_{A}}^{l}-Dv\right\|}_{2}<\delta$$

(5) $$v_{L_{B}}^{l}=\underset{v}{argmin}{\left\|v\right\|}_{0}s.t.{\left\|{\vec{q}}_{L_{B}}^{l}-Dv\right\|}_{2}<\delta$$

where *v* is the sparse coefficient of image *q*, δ is the sparse reconstruction error, and **D** is the pre-trained redundant dictionary, which can be learned using the K-SVD method. K-SVD is an iterative method that alternates between sparse coding of examples based on the current dictionary and updating the dictionary atoms to better fit the data. Further details can be found in [37].

Step c: Sparse reconstruction

The absolute magnitude of the SR coefficients can express the active pixel information in images; thus, the SR coefficients $v_{L_{A}}^{l}$ and $v_{L_{B}}^{l}$ can be selected using the *l*_1_-max rule to obtain the SR coefficient of the fused low-pass layer $v_{F}^{l}$ as given below.

(6) $$v_{F}^{l}=\left\{\begin{array}{cc} v_{L_{A}}^{l} & if{\left\|v_{L_{A}}^{l}\right\|}_{1}>{\left\|v_{L_{B}}^{l}\right\|}_{1}\\ v_{L_{B}}^{l} & otherwise \end{array}\right.$$

A linear representation of $v_{F}^{l}$ that takes advantage of the redundant dictionary **D** yields the content vector component ${\vec{v}}_{F}^{l}$.

(7) $${\vec{v}}_{F}^{l}=Dv_{F}^{l}$$

The operations mentioned above are repeated for each image block of $\{p_{L_{A}}^{l}{\}}_{l=1}^{w}$ and $\{p_{L_{B}}^{l}{\}}_{l=1}^{w}$ to obtain all the content vectors of the fused low-pass layer $\{{\vec{v}}_{F}^{l}{\}}_{l=1}^{W}$, after which each vector is converted into an 8 × 8 image block. Finally, these blocks are rearranged into their original positions to obtain the final fused low-pass layer image $L_{F}^{Q}$.

### 3.2. Fusion of Bandpass Images

Generally, bandpass images, similar to the high-pass component of an image, contain abundant details, including textures, and edges. It is worth noting that the edge and contrast feature is significant for vascular images [38]; thus, the new CSF rule was proposed to fuse the bandpass images adequately considering the neighborhood correlation of pixels and contrast gradient information.

The fusion process used for bandpass images is given by Equation (8):

(8) $$H_{F}^{u,k}(i,j)=H_{A}^{u,k}(i,j)\times M_{A}^{u,k}(i,j)+H_{B}^{u,k}(i,j)\times M_{B}^{u,k}(i,j)$$

where $H_{X}^{u,k}(i,j)$ (*X* = *A*, *B*) represents the bandpass image of *X* at the *u-*th layer and *k-*th direction at position (i,j). $M_{X}^{u,k}(i,j)$ is a decision map of $H_{X}^{u,k}(i,j)$. $H_{F}^{u,k}(i,j)$ is the fused bandpass layer in *u-*th layer in *k-*th direction.

From Equation (8), it may be observed that the fusion performance of the bandpass images depends completely on the accuracy of the corresponding decision maps. In turn, the decision maps can be obtained by comparing the saliency maps of $H_{A}^{u,k}$ and $H_{B}^{u,k}$. Generally, the spatial frequency (SF) can effectively reflect the degree of grayscale variation of an image in a local area, and this variation is related to salient features that can be easily perceived by the human eye. This is especially relevant for optical vascular images, the useful information of which is mainly expressed by salient features. Therefore, the decision map $M_{A}^{u,k}$ can be calculated as given in Equation (9).

(9) $$M_{A}^{u,k}(i,j)=\left\{\begin{array}{cc} 1, & S_{A}^{u,k}(i,j)>S_{B}^{u,k}(i,j)\\ 0, & otherwise \end{array}\right.$$

(10) $$S_{A}^{u,k}(i,j)=\sqrt{(RF_{A}^{u,k}(i,j){)}^{2}+(CF_{A}^{u,k}(i,j){)}^{2}}$$

(11) $$RF_{A}^{u,k}(i,j)=\sqrt{\frac{1}{M\times N}\sum_{i=1}^{M}\sum_{j=2}^{N}[H_{A}^{u,k}(i,j)-H_{A}^{u,k}(i,j-1){]}^{2}}$$

(12) $$CF_{A}^{u,k}(i,j)=\sqrt{\frac{1}{M\times N}\sum_{j=1}^{N}\sum_{i=2}^{M}[H_{A}^{u,k}(i,j)-H_{A}^{u,k}(i-1,j){]}^{2}}$$

According to Equations (10)–(12), $S_{B}^{u,k}(i,j)$ can be obtained similarly. $S_{A}^{u,k}(i,j)$ and $S_{B}^{u,k}(i,j)$ denote the SF images of $H_{A}^{u,k}$ and $H_{B}^{u,k}$ at the pixel (i,j). $RF_{A}^{u,k}(i,j)$ and $CF_{A}^{u,k}(i,j)$ are the vertical and horizontal first-order gradients, respectively, of the pixel (i,j) in theneighborhood of M×N.

The gradient information of the bandpass images can be effectively detected using the SF. However, for the high-frequency components of the vascular images, the main information does not rely only on the gradient structure and requires contrast features to a certain degree (i.e., the information should be both rich and obvious); therefore, the structural edges should be considered. Thus, a novel bandpass image fusion rule that considers both gradient structure and contrast detection is required. To address this problem, a CSF scheme is proposed to optimize $M_{A}^{u,k}$ as follows:

(13) $$\bar{M_{A}^{u,k}}(i,j)=\left\{\begin{array}{cc} 1, & S_{A}^{u,k}(i,j)>\bar{S_{B}^{u,k}}(i,j)\\ 0, & otherwise \end{array}\right.$$

(14) $$\bar{S_{B}^{u,k}}(i,j)=\frac{1}{p\times q}\sum_{i,j\in \Omega }S_{B}^{u,k}(i,j)$$

where $\bar{M_{A}^{u,k}}(i,j)$ represents the improved $M_{A}^{u,k}(i,j)$, $\bar{S_{B}^{u,k}}(i,j)$ is the local contrast of $S_{B}^{u,k}(i,j)$, and p×q represents the neighborhood size. The saliency and contrast characteristics of $H_{X}^{u,k}(i,j)$ can be effectively detected using Equation (14). Furthermore, to correct the inevitable small holes, bumps, and narrow breaks in $\bar{M_{A}^{u,k}}$, adaptive morphological filtering [39] is introduced to process $\bar{M_{A}^{u,k}}$. Then, the intermediate decision map $\bar{M_{A}^{u,k}}$ can be obtained.

(15) $$\bar{M_{A}^{u,k}}(i,j)=bwareaopen(\bar{M_{A}^{u,k}}(i,j),r\times C)$$

where C denotes the area of source image and *r* is the scale factor. All regions smaller than r×C in the binary image $\bar{M_{A}^{u,k}}$ are removed by the ‘*bwareaopen*’ filling filter.

It is worth noting that $\bar{M_{A}^{u,k}}$ may still include some rough or misjudged small areas; in terms of the integrity of the object, $\bar{M_{A}^{u,k}}$ can be further refined through consistency verification [13]. Decision map optimization has been widely used for this purpose in several studies [7]. Thus, the final decision map ${\tilde{M}}_{A}^{u,k}$ can be obtained as follows:

(16) $${\tilde{M}}_{A}^{u,k}(i,j)=\left\{\begin{array}{cc} 1, & if\sum_{(a,b)\in \Theta }\bar{M_{A}^{u,k}}(i+a,j+b)\\ 0, & otherwise \end{array}\right.$$

where Θ is a neighborhood of N×N centered at (i,j), and *a* and *b* represent the horizontal and vertical pixel distances from the center point, respectively. Subsequently, the fused bandpass image ${\tilde{F}}_{H}^{u,k}$ can be generated as shown in Equation (17).

(17) $${\tilde{F}}_{H}^{u,k}(i,j)=H_{A}^{u,k}(i,j)\times {\tilde{M}}_{A}^{u,k}(i,j)+H_{B}^{u,k}(i,j)\times {\tilde{M}}_{B}^{u,k}(i,j)$$

where

(18) $${\tilde{M}}_{B}^{u,k}(i,j)=1-{\tilde{M}}_{A}^{u,k}(i,j)$$

### 3.3. Image Reconstruction

The final fused image *F* can be obtained by performing inverse NSCT [40] on the selected subband images $\left\{L_{F}^{Q},{\tilde{F}}_{H}^{u,k}\right\}$, as given below.

(19) $$F(i,j)=INNSCT({\tilde{F}}_{H}^{u,k}(i,j),L_{F}^{Q}(i,j))$$

## 4. Experiments

The experimental setup is described below in Section 4.1. In Section 4.2, the selection of the parameters is discussed, and the fusion results are presented and discussed in Section 4.3. In Section 4.4, the fusion results for the public dataset are presented.

### 4.1. Experimental Setup

#### 4.1.1. Testing Images

The experimental images were derived from a male mouse (C57BL/6), aged 9 months and weighing 21 g. The mouse was first anesthetized with 0.12 mL of chloral hydrate at a concentration of 0.15 g/mL. Next, the mouse’s ears were depilated with depilatory cream. Then, the anesthetized mouse body was placed on a heating pad at a constant temperature and the mouse ears were attached to the slides with double-sided tape. The animal was treated according to Regulations for the Administration of Affairs Concerning Experimental Animals, which were approved by the State Science and Technology Commission of China, and the Guangdong Province regulations for the care and handling of laboratory animals. For the optical imaging system parameters, the lens magnification and depth of field were set to 1.15 and 0.8 mm, respectively. The camera exposure time and sampling rate were set to 0.45 ms and 42 fps, respectively. The electric zoom lens was moved along the z-direction in 0.3 mm steps on a network of the focal plane, and four groups of multi-focus angiographic images of mouse ears with different resolutions were obtained using the intensity-fluctuation modulation effect.

**Figure 3:**
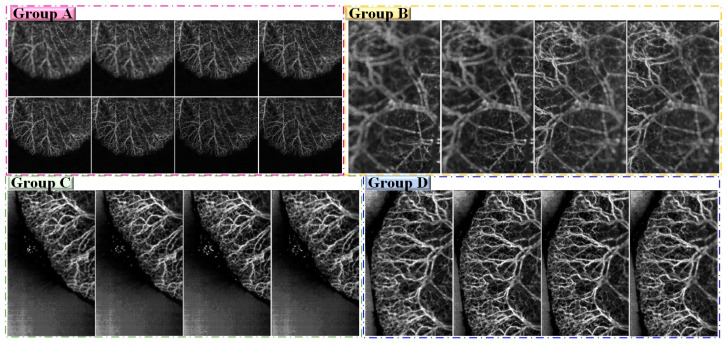
Parts of four groups of source images.

Group A consisted of 30 multi-focus images with a resolution of 726 × 667 pixels, group B consisted of 32 multi-focus images with a resolution of 400 × 667 pixels, group C included 16 multi-focus images with a resolution of 400 × 667 pixels, and group D consisted of 17 multi-focus images with a resolution of 400 × 667 pixels. All the source images were registered. For a fair comparison, these source images in our experiment were the same as in our previous work [34]. Some of them are shown in Figure 3. For the FFOA imaging technique, the range of depth-of-field values is the key factor affecting imaging quality. Some areas of the images obtained in the experiment were within the depth of the field of the lens, whereas others were outside. Owing to the curved and rough surfaces of mouse ears, the blood vessels are primarily distributed in the ear within a volume with a height of approximately 3.3 mm, which makes it difficult to obtain clear images of all blood vessels within a limited depth of field. Figure 3 shows parts of the four groups of multi-focus angiographic images, from which it may be concluded that clear images can be obtained only at the focus position of the lens.

#### 4.1.2. Compared Methods

To verify the effectiveness and superior performance of the proposed method, six state-of-the-art multi-focus fusion algorithms were compared.

(1) Adaptive and gradient joint constraint-based methods (MFF-GAN) [17];
(2) Unified unsupervised network-based methods (U2Fusion) [18];
(3) Swin transformer-based method (SwinFusion) [19];
(4) Simultaneous convolutional sparse approximation-based methods (CSSA) [41];
(5) Nonsubsampled contourlet transform (NSCT-SR) [13];
(6) Contrast pyramid fusion algorithm (CPFA) [34].

The parameters of all the methods compared were in accordance with their published settings.

#### 4.1.3. Testing Platform

In the experiments, CSSA, NSCT, CPFA, and the proposed methods were implemented in MATLAB R2021 with a PC using a Core i7-11700F@2.50 GHz 8-core CPU and 32 GB of RAM. The MFF-GAN, U2Fusion, and SwinFusion methods were implemented in an environment with an AMD Ryzen 54600U with a clock rate of 2.10 GHz with Radeon Graphics.

#### 4.1.4. Quantitative Evaluation Metrics

Quantitative assessment of fused results is a difficult task owing to the unavailability of standard reference images. Furthermore, any given single metric may not reflect the quality of the fused results objectively and adequately. Therefore, to comprehensively and quantitatively evaluate the performance of different methods, six commonly used metrics were used, including (1) standardized mutual information (Q^MI^) [40], (2) Tallis entropy (Q^TE^) [42], (3) nonlinear correlation information entropy (Q^NCIE^) [43], (4) a multi-scale metric (Q^M^) [44], (5) phase consistency (Q^P^) [45], and (6) the fidelity of visual information (VIF) [46].

Among these, VIF is inspired by human perception. Q^MI^ and Q^NCIE^ measure the mutual dependence of two variables. Q^TE^ is a measure of divergence that quantifies the degree of dependence of two discrete variables. Q^M^ measures the edge information retained by the fused image from the source images. Q^P^ is based on spatial frequency, which is also utilized to measure the information of corners and edges preserved in the fused image. VIF measures the fused image in terms of visual quality perspective. Further details on these metrics are available in [47]. Note that larger values indicate better fusion performance.

### 4.2. Discussion of Parameter Selection

For the NSCT used in the proposed method, a four-layer decomposition was applied from coarse to fine in the {2, 2, 2, 2} directions, with “pyrexc” as the pyramid filter and “vk” as the direction filter. It is clear from the results that the four parameters in the proposed scheme had no significant effect on fusion performance. Two parameters slightly affected the fusion performance, including the window size M × N (M = N) in Equation (11), and neighborhood Ω with a size of p × q (p = q) in Equation (14), which can be set as {M = N = 7, p = q = 7}. Furthermore, dictionary **D** in the SR was obtained by pretraining selected high-quality natural images. In addition, two pivotal parameters must be analyzed, including the area elimination share *r* of the morphological filtering in Equation (15) and the window size N of the consistency verification in Equation (16). When one of these parameters was analyzed, the other was fixed. Similar approaches have been used widely to consider the effects of multiple parameters [7].

To determine the most reasonable values of M and N, p and q were fixed at 7, N was fixed at 59, *r* was fixed at 0.05. In contrast, when ascertaining p and q, M = N = 3 was fixed, N was fixed at 59, *r* was fixed at 0.05. Group D (Figure 3) was used as the testing group. The best values for each metric are listed in Table 2. From Table 2, it may be observed that when M = N = 7, the values of Q^MI^, Q^TE^, Q^NCIE^, Q^M^, and Q^P^ were the highest. It is also clear to see that when p = q = 7, the values of Q^MI^, Q^TE^, and VIF exceeded those of the other situations, 7 was chosen as the most reasonable value.

To ascertain the appropriate value of *r*, the parameter N was fixed at 59, and M, N, p, and q are fixed at 7. Again, Group D was employed as testing images. The corresponding quantitative evaluations using different *r* values are presented in Table 2. The value of *r* increased with a step size of 0.005, from 0.02 0.065. It may be observed that the quality of the fused images gradually improved as *r* increased in Table 2. In particular, when *r* was set to 0.05, both Q^MI^ and Q^NCIE^ achieved their maximum values, whereas the Q^TE^, Q^M^, and VIF values ranked second. Based on the data in Table 2, it may be clearly observed that 0.05 was a suitable value of *r* for the proposed method.

To analyze the parameter N in Equation (16), the crucial parameter *r* was fixed at 0.05, M and, N, p, and q were fixed at 7. Using the group C images in Figure 3 as testing images, the optimal range was first roughly determined using large steps, and small steps were then used to locate the range more precisely. The objective evaluations of the different fusion results obtained using different N values are shown in Table 2. As shown in Table 2, starting from N=25, the larger the value of N, the better the fusion performance. When N was set to 59, the performance of the proposed approach was the best in terms of the metrics Q^M^, Q^TE^, Q^NCIE^, Q^M^, and VIF. As N continued to increase, the fusion performance gradually decreased; thus, the results show that it was reasonable to set N to 59 in the experiment.

In summary, the best values of *r* and N were {M = N = 7, p = q = 7, r = 0.05, N = 59}.

### 4.3. Fusion Results and Discussion

#### 4.3.1. Subjective Evaluation

**Figure 4:**
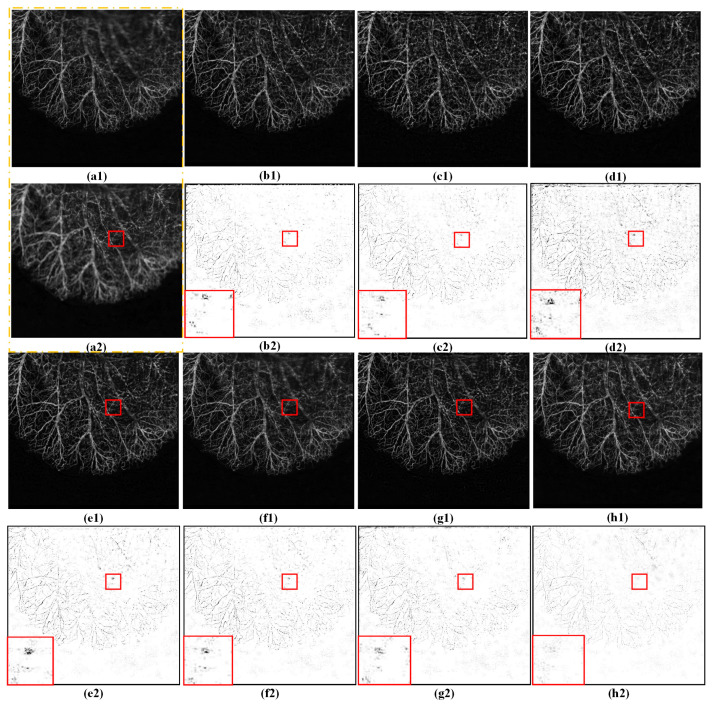
Fusion results produced by different methods. (**a1**,**a2**) are two randomly selected source images of group A; (**b1**–**h1**) show the fused images obtained by MFF-GAN, U2Fusion, SwinFusion, NSCT, CSSA, CPFA, and the proposed method, respectively; and (**b2**–**h2**) are the difference images obtained from the corresponding fused images and (**a2**), respectively. The red box is a magnification of the local details.

**Figure 5:**
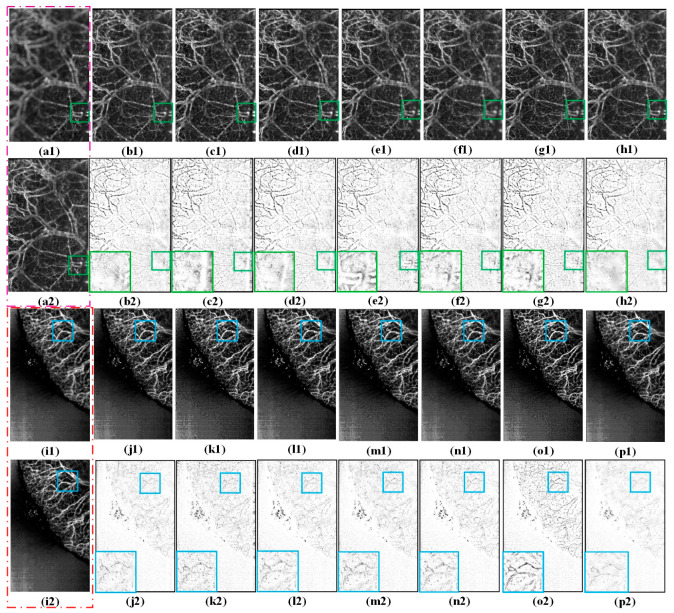
Fusion results produced by different methods. (**a1**,**a2,i1**,**i2**) are randomly selected source images of groups B and C, respectively, whereas (**b1**–**h1**,**j1**–**p1**) are the fused images obtained using the MFF-GAN, U2Fusion, SwinFusion, NSCT, CSSA, and CPFA models and the proposed method. (**b2**–**h2**) are the difference images obtained by subtracting the corresponding fused image from the source image of (**a1**), respectively, and (**j2**–**p2**) are the difference images obtained by subtracting the corresponding fused image from (**i2**). The green and blue boxes are enlargements of two local details.

To better observe the different fusion results in terms of the subjective visual perception, the corresponding difference images are shown in the Figure 4 and Figure 5, respectively. Each difference image was obtained by subtracting a source image from the corresponding fused image. For focused regions, the closer the residuals in the difference image are to zero, the better the quality of the fused result. Considering the length of this study, only three groups of fusion examples are provided.

Figure 4 shows the fused results produced by the different methods from the source images in group A. For a better comparison, the areas framed with pink squares in the difference images are enlarged in Figure 4(b2–h2). Some “cloud-like” artifacts appeared in the compared methods, indicating that a few unfocused pixels in the source images were transferred to the fused images. The CSSA, CPFA, and NSCT methods have less residual information in the difference images (see Figure 4(e2–g2)), whereas MFF-GAN, U2Fusion, and SwinFusion (see Figure 4(b2–d2)) have the most residual information. Unfocused regions also appear near the top of the fused image obtained by CSSA (see Figure 4(f1)). In contrast, little residual information and few visual artifacts appeared in the results of the proposed method (Figure 4(h2)). This demonstrates that the proposed method is superior to the other fusion methods in terms of visual effects.

The source image groups B and C are the FFOA multi-focus images of mouse ears, and the corresponding fusion results generated by different methods are shown in Figure 5. For convenience of observation, the local regions of Figure 5(b2–h2,j2–p2) are enclosed by colored boxes and enlarged at their respective top- and bottom-right corners.

From these regions, it may be clearly observed that the fusion results obtained using the MFF-GAN, NSCT, CSSA, and CPFA (see Figure 5(b1,e1–g1,j1,m1–o1)) lost some important contour details of the source images, and a blurring effect appeared in the fused results. In other words, the MFF-GAN, NSCT, CSSA, and CPFA methods could not effectively transfer the distinct vascular features of the source images into the fused image. Although the fused images obtained by U2Fusion and SwinFusion exhibited higher sharpness than those obtained by the MFF-GAN, NSCT, CSSA, and CPFA methods, U2Fusion (see Figure 5(c1)) was less effective at preserving edge details. Note that the difference images of SwinFusion and U2Fusion (Figure 5(c2,d2,k2,l2)) show that some focused information was not fully transferred to the fused images. In comparison, less residual information remained in the difference images generated by the proposed approach (see Figure 5(h2,p2)), more details and texture features were retained, and the border region between the focused and unfocused images was complete and natural (Figure 5(h1,p1)) compared to the other fusion results. Thus, the proposed method can extend the depth-of-field range of FFOA imaging by removing the depth-of-field limitation and clarifying blurred vessels.

#### 4.3.2. Objective Evaluation

**Figure 6:**
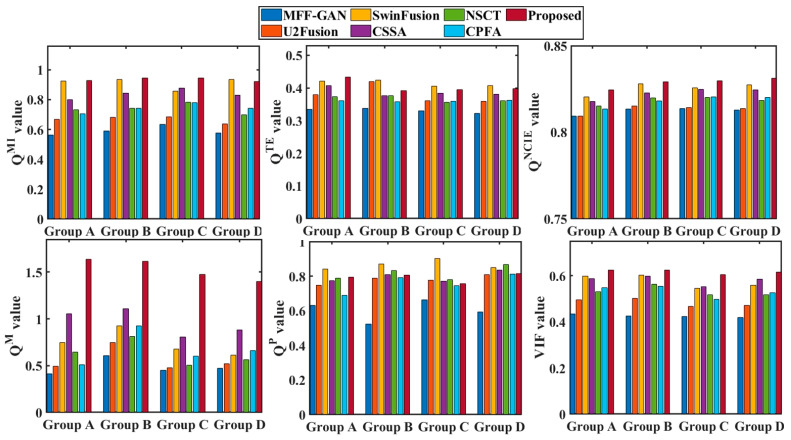
Quantitative evaluations of different methods for four groups of the source images.

**Figure 7:**
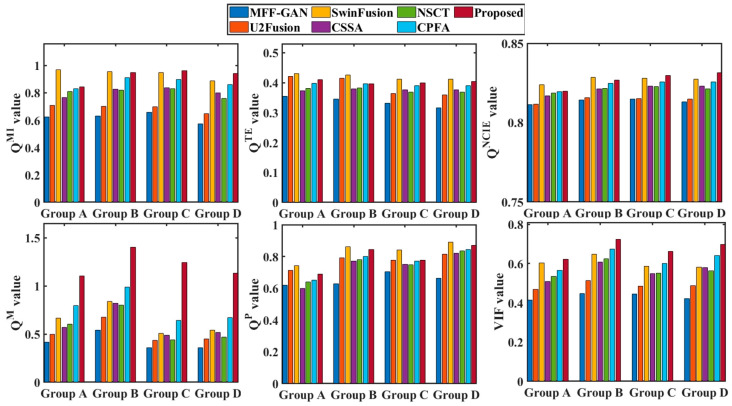
Quantitative evaluations of the intermediate and final fused images obtained by different methods for four groups of source images.

In addition to the above subjective visual comparisons, the metrics Q^MI^, Q^TE^, Q^NCIE^, Q^M^, Q^P^, and VIF were used for quantitative evaluations. Figure 6 shows the objective evaluations of the final fused results for each Group A images in Figure 3, whereas Figure 7 shows the average performance of the quantitative evaluations of all the fusion results (e.g., as there were 30 source images in Group A, there were a total of 29 fusion processes).

**Figure 8:**
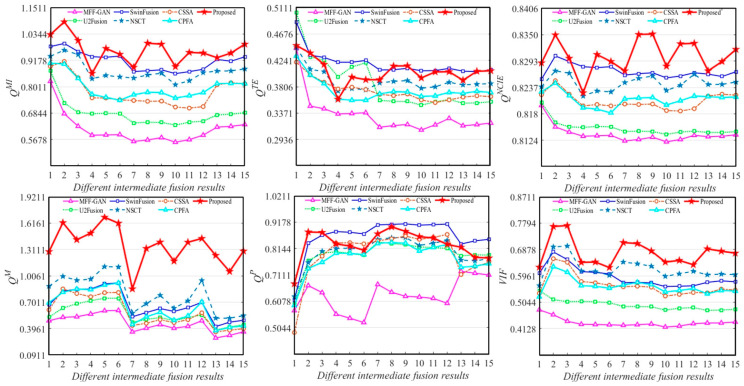
Quantitative evaluations of 15 randomly selected intermediate fusion results.

Figure 8 show the averaging quantitative evaluations of the 15 intermediate results for source images; these fused results were generated by MFF-GAN, U2Fusion, SwinFusion, NSCT, CSSA, CPFA, and the proposed method.

From Figure 6 and Figure 7, it may be observed that SwinFusion, NSCT, and the proposed method obtained relatively high scores. Among them, the proposed method performed best. As shown in Figure 6, although the SwinFusion method exhibited the highest score in terms of the Q^P^ and Q^TE^ metrics for image groups B and C, the best Q^P^ for group A, and the best Q^MI^ and Q^TE^ for group D, the other metrics were relatively moderate. As shown in Figure 7, the SwinFusion method performed better for the fusion of groups A and B, although the proposed method still performed better overall. Furthermore, the performance of the U2Fusion, CPFA, and CSSA methods was poor, and the MFF-GAN method exhibited a relatively poor performance. In contrast, the proposed method outperformed the other six methods in terms of quantitative evaluation, indicating that it performed well in terms of useful information retention, source image feature retention, and human perception realism retention.

As may be observed from Figure 8, the results of the Q^TE^ and Q^P^ metrics for SwinFusion and the proposed method are similar, indicating that both methods were able to retain the useful feature information of the source images better. The value of the MFF-GAN and U2Fusion methods under the six evaluation metrics was the lowest compared to those of the other methods, which exhibited poor fusion performance. The CSSA, NSCT, and CPFA methods were relatively competitive, but there was still a gap in their fusion performances compared with the proposed method. In addition, the results of the proposed method far exceeded those of the other methods using the Q^M^ metric, indicating the superior image feature extraction capability of the proposed method.

The results of the objective evaluation in terms of Q^MI^, Q^TE^, Q^NCIE^, Q^M^, Q^P^, and VIF acquired using the different methods are in line with the results of the subjective visual analysis overall. The effectiveness and reliability of the proposed scheme are clearly validated based on a subjective visual comparison of the qualitative evaluations.

### 4.4. Extended to More Public Datasets

To confirm the effectiveness and superiority of the proposed method, extensive experiments were also conducted on the public multi-focus Lytro dataset, which contains 20 pairs of source images.

Six highly convictive image fusion metrics, including Q^MI^, Q^TE^, Q^NCIE^, Q^M^, Q^P^, and VIF were used to quantitatively assess the fused results produced by the different methods. From Table 3, it may be observed that the Q^TE^ value of U2Fusion and the Q^M^ value of CPFA were slightly higher than those of the proposed method; however, the fusion performance of the proposed method was nonetheless notably better overall. It may be easily observed that MFF-GAN and SwinFusion models performed better than U2Fusion and CSSA, whereas the gap between the performance of the second method and that of the proposed method was larger than that between the second and the last methods. Furthermore, the proposed method performed well in four aspects: Q^MI^, Q^NCIE^, Q^P^, and VIF ranked first among the compared methods. Above all, the results show that the performance of the proposed method was considerably superior to that of existing state-of-the-art multi-focus image fusion methods in terms of clarity and contrast.

### 4.5. Computational Complexity

The time required for image fusion with the seven methods is listed in Table 4, and each time was ranked. The CPFA method required the shortest time owing to its pyramid-based fusion rule. The CSSA method is based on simultaneous sparse approximation with the longest running time. The computational efficiencies of the MFF-GAN, U2Fusion, and NSCT methods were relatively high. SwinFusion has medium fusion efficiency owing to the design of the cross-regional distance learning model. The time consumed by the proposed method was primarily during the execution of the SR model. We consider that the computational efficiency of the proposed method would be significantly improved by using the C++ programming langue or GPU hardware.

## 5. Conclusions

In this study, an FFOA imaging system based on the intensity-fluctuation modulation effect was developed, the frequency distribution of dynamic scatter and static scatter is obtained by fast Fourier transform of the original scatter signal, and the two frequency domain signals are separated to achieve the extraction of dynamic blood flow information. Meanwhile, a novel multi-focus image fusion method based on NSCT and CSF was proposed to fuse multi-depth-of-field FFOA images. Through this method, the defocusing problem of FFOA images caused by the limited depth of field of the optical lens and the uneven surface thickness of biological samples can be solved. The source images were decomposed into low-pass and bandpass images using NSCT. Subsequently, the proposed diffusion rules of the SR and CSF were described according to different features of the two subbands. Finally, the fused low-pass and bandpass images were inverted using NSCT to obtain the final fused image. This method fully considers the energy information in lowpass images. Furthermore, the neighborhood correlation and gradient information of the pixels in the bandpass images are considered to preserve the energy, detail, and structure of the source images effectively. The results of the experimental evaluation clearly demonstrate the superiority of the proposed method compared with the other six popular multi-focus image fusion methods in terms of subjective visual comparisons and quantitative evaluation metrics.

This work provided effective technical support for the extension of the depth of an optical lens. It also has essential applications in bioimaging and medical diagnosis. Although the proposed method performed well on multi-focus image fusion tasks, considerable room for improvement remains in terms of its strategy for fusing boundary regions. In future work, the designed algorithm will be further optimized to better handle the boundary problem and will be improved to enhance its robustness in fusing noise-disturbing and misregistered images.

## Figures and Tables

**Table 1:** Summary of related works mentioned above.

Algorithms	Methods	References
Spatial domain-based methods	Scheme based on difference-of-Gaussians	[25]
	Joint guided image filtering-based approach	[9]
	Random-walk estimation-based method	[10]
	Multi-scale shearing nonlocal guided averaging filter-based scheme	[11]
Deep learning-based method	CNN with integrated learning-based scheme	[16]
	Joint adaptive and gradient constraints-based method	[17]
	Unsupervised fusion network-based method	[18]
	Swin transformer-based method	[19]
Multi-scale transform-based methods	Hessian matrix-based method	[14]
	Domain transform filtering-based approach	[29]
	Adaptive energy choosing-based scheme	[30]
	Neighbor energy activity operator-based scheme	[31]
	Embedding bilateral filter in least squares-based method	[32]
	NSCT-based method	[13]
	Residual removal technique-based approach	[7]
Sparse representation-based methods	Taylor expansion-based approach	[20]
	Joint fusion and super-resolution scheme	[21]
	Multi-scale sparse representation-based image fusion scheme	[22]

**Table 2:** Quantitative evaluations of fused images with different parameters, best values are bolded.

Metrics	Parameter M						
	3	5	7	9	11	13	15
Q^MI^	0.9371	0.9347	**0.9393**	0.9289	0.9371	0.9371	0.9371
Q^TE^	0.4012	0.4007	**0.4018**	0.3998	0.4012	0.4012	0.4012
Q^NCIE^	0.8324	0.8323	**0.8326**	0.8317	0.8324	0.8324	0.8324
Q^M^	1.4355	1.4309	**1.4437**	1.3900	1.4355	1.4355	1.4355
Q^P^	0.8187	0.8180	**0.8193**	0.8183	0.8187	0.8187	0.8187
VIF	**0.6179**	0.6175	0.6177	0.6159	**0.6179**	**0.6179**	**0.6179**
Metrics	Parameter p						
	5	7	9	11	13	15	17
Q^MI^	0.9343	**0.9371**	0.9343	0.9345	**0.9371**	**0.9371**	0.9081
Q^TE^	0.4009	**0.4012**	0.4007	0.4009	**0.4012**	**0.4012**	0.3907
Q^NCIE^	0.8322	0.8324	0.8322	0.8322	0.8324	0.8324	**0.8332**
Q^M^	1.4308	1.4355	1.4374	**1.4418**	1.4396	1.4396	0.9644
Q^P^	0.8179	0.8187	0.8180	**0.8192**	0.8189	0.8189	0.8073
VIF	0.6187	**0.6179**	0.6175	0.6176	0.6176	0.6176	0.6176
Metrics	Parameter r						
	0.035	0.04	0.045	0.05	0.055	0.06	0.065
Q^MI^	0.9124	0.9190	0.9283	**0.9350**	0.9316	0.9336 (2)	0.9333
Q^TE^	0.3951	0.3971	0.3987	0.4002 (2)	0.3999	0.4001	**0.4005**
Q^NCIE^	0.8305	0.8310	0.8318	**0.8323**	0.8321	0.8322 (2)	0.8322 (2)
Q^M^	1.3693	1.3815	1.4052	1.4395 (2)	1.4246	1.4341	**1.4434**
Q^P^	0.8192	0.8182	0.8185	0.8167	0.8172	0.8163	0.8167
VIF	0.6141	0.6143	0.6158	0.6167 (2)	**0.6168**	0.6165	0.6162
Metrics	Parameter N						
	45	55	57	59	61	63	65
Q^MI^	0.9014	0.9041	0.9342	**0.9371**	0.9340	0.9336	0.9003
Q^TE^	0.3891	0.3906	0.4008	**0.4012**	0.4005	0.4005	0.3894
Q^NCIE^	0.8296	0.8298	0.8322	**0.8324**	0.8322	0.8322	0.8295
Q^M^	1.3504	1.3702	1.4258	**1.4355**	1.4349	1.4268	1.3454
Q^P^	0.8208	**0.8270**	0.8168	0.8187	0.8172	0.8168	0.8209
VIF	0.6148	0.6170	0.6169	**0.6179**	0.6169	0.6168	0.6145

**Table 3:** Quantitative evaluations on Lytro dataset, best values are bolded.

Source Images	Metrics	MFF-GAN	U2Fusion	SwinFusion	CSSA	CPFA	Proposed
Lytro dataset	Q^MI^	0.8201(3)	0.7302	0.8400(2)	0.7890	0.1763	**0.9924**
	Q^TE^	0.3863	**0.4116**	0.3962(3)	0.3894	0.2263	0.4087(2)
	Q^NCIE^	0.8251(3)	0.8212	0.8258(2)	0.8242	0.8054	**0.8337**
	Q^M^	0.4878(3)	0.3790	0.4798	0.4865	**2.6280**	2.0777(2)
	Q^P^	0.7931(3)	0.7461	0.8038(2)	0.7871	0.0562	**0.8626**
	VIF	0.4901	0.4625	0.5475(2)	0.5211(3)	0.1706	**0.6253**

**Table 4:** Runtime for different methods.

Methods	MFF-GAN	U2Fusion	SwinFusion	CSSA	NSCT	CPFA	Proposed
Time/s	3.91(3)	0.66(2)	74.00(6)	142.49(7)	5.14(4)	**0.10**	51.56(5)

## Data Availability

Data sharing not applicable.
